# Reporting transparency and completeness in trials: Paper 4 - reporting of randomised controlled trials conducted using routinely collected electronic records – room for improvement

**DOI:** 10.1016/j.jclinepi.2021.09.011

**Published:** 2022-01

**Authors:** Stephen J. McCall, Mahrukh Imran, Lars G. Hemkens, Kimberly Mc Cord, Linda Kwakkenbos, Margaret Sampson, Sena Jawad, Merrick Zwarenstein, Clare Relton, Sinéad M. Langan, David Moher, Ole Fröbert, Brett D. Thombs, Chris Gale, Edmund Juszczak, on behalf of CONSORT Extension for Trials Conducted Using Cohorts and RoutinelyCollected Data Group

**Affiliations:** a Nuffield Department of Population Health, National Perinatal Epidemiology Unit Clinical Trials Unit, University of Oxford, Oxford, United Kingdom; b Center for Research on Population and Health, American University of Beirut, Ras Beirut, Lebanon; c Lady Davis Institute for Medical Research, Jewish General Hospital, Montreal, Quebec, Canada; d Basel Institute for Clinical Epidemiology and Biostatistics, Department of Clinical Research, University Hospital Basel, University of Basel, Basel, Switzerland; e Behavioural Science Institute, Clinical Psychology, Radboud University, Nijmegen, the Netherlands; f Library Services, Children's Hospital of Eastern Ontario, Ottawa, Canada; g Neonatal Medicine, School of Public Health, Faculty of Medicine, Imperial College London, London, United Kingdom; h Department of Family Medicine, Schulich School of Medicine and Dentistry, Western University, London, Ontario, Canada; i Institute for Clinical Evaluative Sciences, Toronto, Canada; j Centre for Clinical Trials and Methodology, Barts Institute of Population Health Science, Queen Mary University, London, United Kingdom; k Faculty of Epidemiology and Population Health, London School of Hygiene and Tropical Medicine, London, United Kingdom; l Centre for Journalology, Clinical Epidemiology Program, Ottawa Hospital Research Institute, Ottawa, Ontario, Canada; m Department of Cardiology, Faculty of Health, Örebro University, Örebro, Sweden; n Department of Psychiatry, McGill University, Montreal, Quebec, Canada; o Department of Epidemiology, Biostatistics and Occupational Health,McGill University, Montreal, Quebec, Canada; p Department of Medicine, McGill University, Montreal, Quebec, Canada; q Department of Educational and Counselling Psychology, McGill University, Montreal, Quebec, Canada; r Department of Psychology, McGill University, Montreal, Quebec, Canada; s Biomedical Ethics Unit, McGill University, Montreal, Quebec, Canada; t Nottingham Clinical Trials Unit, Applied Health Research Building, University of Nottingham, Nottingham, United Kingdom

**Keywords:** CONSORT-ROUTINE, Extension, Routinely, Collected, Health, Data

## Abstract

**Objective:**

To describe characteristics of randomized controlled trials (RCTs) conducted using electronic health records (EHRs), including completeness and transparency of reporting assessed against the 2021 CONSORT Extension for RCTs Conducted Using Cohorts and Routinely Collected Data (CONSORT-ROUTINE) criteria.

**Study Design:**

MEDLINE and Cochrane Methodology Register were searched for a sample of RCTs published from 2011–2018. Completeness of reporting was assessed in a random sample using a pre-defined coding form.

**Results:**

Of the 183 RCT publications identified, 122 (67%) used EHRs to identify eligible participants, 139 (76%) used the EHR as part of the intervention and 137 (75%) to ascertain outcomes. When 60 publications were evaluated against the CONSORT 2010 item and the corresponding extension for the 8 modified items, four items were 'adequately reported' for most trials. Five new reporting items were identified for the CONSORT-ROUTINE extension; when evaluated, one was ‘adequately reported’, three were reported ‘inadequately or not at all’, the other ‘partially’. There were, however, some encouraging signs with adequate and partial reporting of many important items, including descriptions of trial design, the consent process, outcome ascertainment and interpretation.

**Conclusion:**

Aspects of RCTs using EHRs are sub-optimally reported. Uptake of the CONSORT-ROUTINE Extension may improve reporting.

**What is new?**

• This review of publications of randomized controlled trials (RCTs) conducted using electronic health records (EHR) finds that EHR were used in different ways: approximately two-thirds of RCTs used the EHR to identify participants, and three-quarters used the EHR to ascertain the outcomes or as part of delivering the intervention.
• The reporting quality for RCTs conducted using EHRs was assessed using the 2021 CONSORT Extension for RCTs Conducted Using Cohorts and Routinely Collected Data (CONSORT-ROUTINE) checklist; reporting was inadequate for newly developed items specific to EHR trials. Most publications did not accurately describe the EHR used or report on the accuracy (e.g., completeness of record linkage) and validity of trial data (e.g., adjudication for outcomes) derived from the EHR.
• In a sample of EHR trials, reporting quality was also inadequate for key trial components covered by the CONSORT 2010 statement for RCTs, including the description of the trial design, the mechanism for concealing allocation and the source and role of the funder.
• The 2021 CONSORT Extension for RCTs Conducted Using Cohorts and Routinely Collected Data (CONSORT-ROUTINE) checklist will provide a benchmark to help improve and guide complete and transparent reporting.

## 1 Introduction

Electronic health records (EHR) are digital versions of medical records. Their primary use is clinical documentation of a patient's medical history [1]. These data are collected prospectively and can provide a comprehensive longitudinal record of a patient's health. The availability of sequential electronic records provides an opportunity for the secondary use of these data for research purposes. EHR data have been widely used for observational studies and are increasingly being used in randomized controlled trials (RCTs) [2].

Common difficulties encountered when conducting RCTs include slow recruitment [3] and extensive monitoring and regulatory requirements [4] which increase the resources required to complete them. Large-scale RCTs are required to definitively answer research questions by robustly generating precise estimates of treatment effects and harms [5,6]. In response to these challenges, new approaches to conducting RCTs have been developed, including using existing sources of data, such as cohorts and routinely collected data (electronic health records, administrative databases and registries). EHRs offer the opportunity to conduct RCTs efficiently through automatic systems which can identify potential participants, assess their eligibility, record consent, randomize the participants, collect trial data and in some situations, deliver trial interventions [7]. These potential gains in efficiency should, in theory, facilitate recruitment from more sites, and include hard to reach patient populations, thus allowing trialists to recruit a greater proportion of eligible patients. Therefore, RCTs using EHRs, particularly those that are closely integrated into care pathways, have the potential to increase efficiency, reduce cost, and generate results that are widely applicable.

The Consolidated Standards of Reporting Trials (CONSORT) 2010 Statement checklist is a 25-item instrument that was established to facilitate transparent and complete reporting of RCTs [8]. RCTs published within journals that endorse the CONSORT statement are better reported than those published in journals that do not [9]. The CONSORT 2010 statement was designed for parallel-group trials, and extensions have been created to meet the reporting requirements of other RCT designs [10], [11], [12]. This review forms part of a larger project developing a CONSORT extension for RCTs conducted using cohorts and routinely collected data (including EHRs, administrative databases and registries) [13]. It is not known how well RCTs that use EHRs are reported. The purpose of this review was two-fold; firstly, to describe characteristics of RCTs conducted using EHRs published January 2011–March 2018, after the publication of the 2010 CONSORT statement [8], and, secondly, to describe the completeness and transparency of reporting of a random subset of trials conducted using EHRs in relation to reporting items developed for the 2021 CONSORT Extension for RCTs Conducted Using Cohorts and Routinely Collected Data (CONSORT-ROUTINE) [14].

## 2 Methods

This review examines reporting in trials using EHR data. The protocol is accessible on open science framework: https://osf.io/p6wa4/.

### 2.1 Inclusion and exclusion criteria

This review examined a subset of trials conducted using EHRs identified in a scoping review that supported the development of the 2021 CONSORT Extension for RCTs Conducted Using Cohorts and Routinely Collected Data (CONSORT-ROUTINE) [15]. Eligible publications reporting RCTs had to either (i) describe using an EHR to identify potentially eligible participants (for further screening), or use the EHR to automatically identify (and facilitate recruitment of eligible) participants; or (ii) describe using an EHR to collect trial process(es) or clinical outcome data; or (iii) describe using the EHR for delivering the intervention (e.g., using clinical decision support systems embedded within the EHR), or any combination of the three uses. Methodological reviews, protocols and commentaries and studies that only assessed cost effectiveness were excluded.

### 2.2 Search strategy and publication selection

A search was performed to identify publications describing methodology, trial protocols or main publications specifically for RCTs that were conducted using EHRs. Ovid MEDLINE Epub Ahead of Print, In-Process & Other Non-Indexed Citations, Ovid MEDLINE Daily and Ovid MEDLINE and EBM Reviews–Cochrane Methodology Registry (Final issue, third Quarter 2012) were searched from January 2007 to March 2018 (Cochrane Methodology Register up to last update in July 2012). A MEDLINE search strategy was developed by a research librarian (MS) with input from the project team and were peer-reviewed using the Peer Review of the Electronic Search Strategy (PRESS) standard [16]. The strategy was then adapted for the Cochrane Library Methodology Register (see Appendix 1 for search strategy). References were imported into Refworks, and duplicates were removed. The references were then imported into the systematic review software DistillerSR (Evidence Partners, Ottawa, Canada, [17]. A coding manual for inclusion and exclusion is available in Appendix 2.

Titles and abstracts were screened independently by two reviewers. A ‘liberal accelerated’ method (where titles and abstracts are screened by one reviewer and only excluded publications are screened by a second reviewer) was used to identify publications for inclusion for full-text review [18]. This was done in random order so that reviewers were blind to whether the other reviewer had already made a decision on any given title and abstract. Full texts were screened independently by two reviewers, and discordances were resolved in consultation with a third reviewer.

### 2.3 Data extraction

Data were extracted into a pre-defined form using the DistillerSR system. Items extracted from each trial publication included: research question, unit of randomization (cluster or individual), setting, location, disease of interest, use of EHR in the trial, intervention, type of EHR related interventions, comparator, primary outcome, whether primary outcome was assessed using EHR, country where the RCT was conducted and the sample size (number of clusters and/or participants randomized) [19]. These items are presented for all trials and separately for cluster vs. individually randomized RCTs. The initial data extraction was completed by one person and was independently validated by another using the Distillers Quality Control function.

Because of the large number of EHR trials identified (183) and for feasibility, a simple random sample of sixty publications of the included full-text publications were evaluated for reporting characteristics; randomization was performed using the ‘random study identifier’ function within DistillerSR. A sample size of 60, assuming that 50% of publications exhibit a certain level of reporting, provides a precision (95% confidence interval) of ± 13.2%.

### 2.4 Evaluation of completeness and transparency of reporting

We evaluated the completeness and transparency of all new and modified items included in the 2021 CONSORT Extension for RCTs Conducted Using Cohorts and Routinely Collected Data (CONSORT-ROUTINE) [14]. For modified items, we first assessed reporting against the CONSORT 2010 checklist item. Then, we assessed only the modified portion of the item. We did this to determine whether any cases of inadequate reporting lacked information specific to the 2021 CONSORT Extension for RCTs Conducted Using Cohorts and Routinely Collected Data (CONSORT-ROUTINE), specifically, or if reporting was inadequate based on CONSORT guidance available at the time of publication.

The reporting of items was categorized into ‘adequately reported’, ‘partially reported’, ‘inadequately or not reported’ and ‘not applicable’. A coding manual was devised to ensure objective assessment of reporting (Appendix 3) based upon similar previous reviews [20,21]. The data extraction rules and coding manual were piloted on five publications by four authors to clarify wording and calibrate agreement between reviewers. A second reviewer validated the final assessment of completeness and transparency of reporting; disagreements were resolved by a third reviewer. Reviewers were not blinded to author or journal names due to lack of evidence that this reduces bias [9,22].

Results were synthesized by totaling the number and percentage of publications adequately, partially, and inadequately or not at all for each item.

## 3 Results

**Fig 1:**
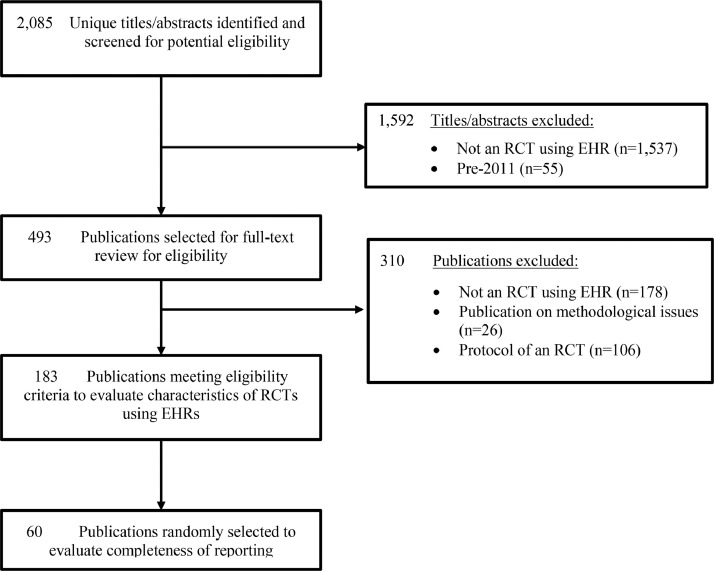
Flow diagram of publication selection process – trials conducted using electronic health records.

The database searches identified 2,085 unique titles and abstracts for review, of which 1,537 were excluded after review of titles and abstracts and 365 after full-text review, leaving 183 publications that met inclusion criteria and were included in the description of RCTs using EHRs. Of these, 60 were randomly selected for review of reporting completeness and transparency (See Fig 1 for selection diagram and Appendix 4 for references).

### 3.1 Characteristics of eligible trial publications

**Fig 2:**
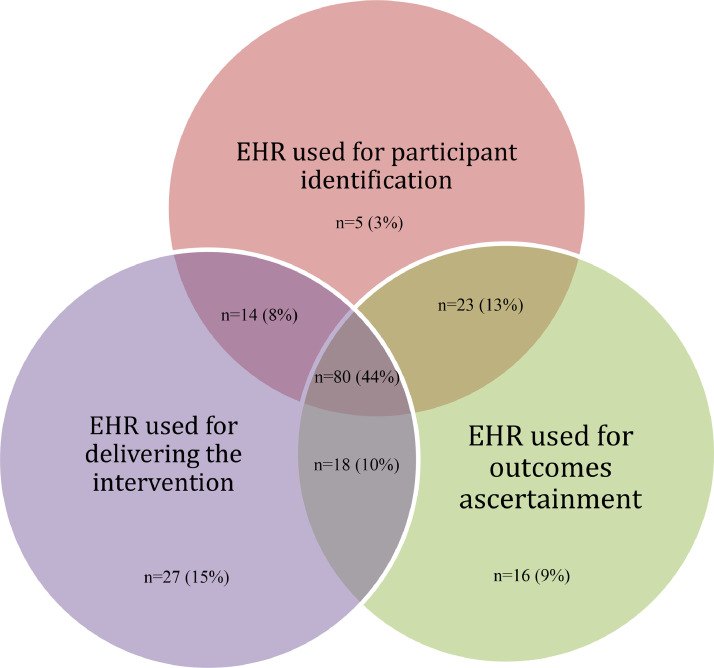
Description of how the electronic health records were used within the total sample of published RCTs meeting eligibility criteria (*n* =183).

Of the 183 RCT publications included, 122 (67%) reported identifying participants using the EHR, 139 (76%) used the EHR for delivering the intervention and 137 (75%) for outcome ascertainment; 80 (44%) used the EHR to perform all three functions (Fig 2).

Characteristics of the 183 RCTs using EHRs are presented in Table 1. The majority were conducted in North America (*n* = 143, 78%), or set in primary care, accident & emergency or outpatient clinics (*n* = 144, 79%). The most common speciality or thematic of interest was internal or general medicine (*n* = 112, 61%). The most common interventions tested were implementation interventions using guideline or reminder-based interventions (*n* = 87, 48%) to improve evidence based care; in trials where the EHR was used for delivering the intervention, the intervention was most commonly a clinical decision support tool (N=91/139, 65%).

**Table 1:** Characteristics of trials conducted using electronic health records.

	*Number (%) of cluster randomised trials (n = 84)*		*Number (%) of individually randomised trials (n = 99)*		*Total (n = 183)*	
*Setting*						
Primary Care, Accident & Emergency or Outpatient	71	(84.5)	73	(73.7)	144	(78.7)
Inpatient	9	(10.7)	16	(16.2)	25	(13.7)
Other	4	(4.8)	10	(10.1)	14	(7.7)
*Country*						
North America	65	(77.4)	78	(78.8)	143	(78.1)
Europe	18	(21.4)	12	(12.1)	30	(16.4)
Rest of the World	1	(1.2)	9	(9.1)	10	(5.5)
*Speciality or Thematic of Interest*						
Internal Medicine or General Medicine	53	(63.1)	59	(59.6)	112	(61.2)
Mental Health or Neurology	3	(3.6)	7	(7.1)	10	(5.5)
Vaccinations	8	(9.5)	7	(7.1)	15	(8.2)
Paediatrics	4	(4.8)	8	(8.1)	12	(6.6)
Behavioural Risk Factors^a^	8	(9.5)	9	(9.1)	17	(9.3)
Other	8	(9.5)	9	(9.1)	17	(9.3)
*Intervention*						
Guideline or Reminder-based	43	(51.2)	44	(44.4)	87	(47.5)
Other	29	(34.5)	46	(46.5)	75	(41.0)
Screening	12	(14.3)	9	(9.1)	21	(11.5)
*Comparator*						
Active Comparison Group	12	(14.3)	20	(20.2)	32	(17.5)
Usual care	71	(84.5)	74	(74.7)	145	(79.2)
Placebo	0	(0.0)	1	(1)	1	(0.5)
Unclear	1	(1.2)	4	(4)	5	(2.7)
*Outcome*						
Mortality, Disease Occurrence or Composite	4	(4.8)	5	(5.1)	9	(4.9)
No Primary Outcome	4	(4.8)	12	(12.1)	16	(8.7)
Other	19	(22.6)	14	(14.1)	33	(18.0)
Self-reported	7	(8.3)	22	(22.2)	29	(15.8)
Surrogate	14	(16.7)	17	(17.2)	31	(17.0)
Up Take of Treatment or Service	36	(42.9)	29	(29.3)	65	(35.6)
*EHR used for Intervention*^b^						
Clinical Decision Support	59	(70.2)	32	(32.3)	91	(49.7)
EHR not used for Intervention	19	(22.6)	25	(25.3)	44	(24.0)
Other	1	(1.2)	5	(5.1)	6	(3.3)
Personal Health Record	3	(3.6)	20	(20.2)	23	(12.6)
Telehealth	2	(2.4)	17	(17.2)	19	(10.4)
*EHR for used for Primary Outcome(s)*^b^						
No/Not Clear	20	(23.8)	45	(45.5)	65	(35.5)
Yes	64	(76.2)	54	(54.5)	118	(64.5)
*Sample size*						
Number of Clusters (median and IQR)	27	(15-56)				
Number of Participants (median and IQR)	4,447	(613-20,904)	415	(123-2,239)		
Total Number of Participants	2,311,604		302,055			

a Includes: Smoking, obesity, alcohol or opioid use.

b Definitions were adapted from Hemkens and Mc Cord, 2019, CMAJ (7)

There were 84 cluster RCTs and 99 individually randomized trials. The proportion of RCTs that tested a clinical decision support tool differed between cluster RCTs (59 of 84, 70%) and individually randomized trials (32 of 99, 32%). Overall, the most common outcome was the uptake of a treatment or service (cluster RCT, 36 of 84, 43% vs. individually randomized RCT, 29 of 99, 29%). Most trials used the EHR to identify the primary outcome (64 of 84, 76%, cluster RCT vs. 54 of 99, 55%, individually randomized RCT). For cluster trials that used EHR systems, the median number of participants were 4,447 (interquartile range [IQR] 613–20,904) with a median of 27 clusters (IQR 15–56); for individually randomized trials, the median number of participants was 415 (IQR 123–2,239).

### 3.2 Baseline assessment of completeness and transparency of reporting

*Results for all included trials are available at* https://osf.io/zjv7k/

CONSORT 2010 items with Modifications in CONSORT-ROUTINE: When publications were evaluated against the original version of the eight CONSORT 2010 items that were modified in the extension, four items were ‘adequately reported’ for most trials, including *Eligibility criteria,* 83%, *Outcome definition,* 73%, *Participant flow,* 75% and *Interpretation,* 80% (Table 2). Only 43%, 28% and 42% ‘adequately reported’ *structured summary (abstract), Trial design* and *Funding*, respectively. *Allocation concealment mechanism* was the most poorly reported item; 60% reported this ‘inadequately or not at all’.

**Table 2:** Completeness and transparency of reporting for 60 trial publications sampled that used the EHR to identify participants and outcomes (includes only new and modified CONSORT 2010 items)^a^

	*Item*^b^	*CONSORT 2010 Items, CONSORT-ROUTINE modifications, and new CONSORT-ROUTINE items*	*n = 60*			
			*Adequately reported n(%)*	*Partially reported n (%)*	*Inadequately or Not reported n(%)*	*Not applicable n (%)*
*Title and abstract*						
	1^b^	CONSORT 2010: Structured summary of trial design, methods, results, and conclusions (for specific guidance see CONSORT for abstracts).	26 (43%)	34 (57%)	0 (0%)	-
		Modified CONSORT-ROUTINE: Structured summary of trial design, methods, results, and conclusions (for specific guidance see CONSORT for abstracts). *Specify that a cohort or routinely collected data were used to conduct the trial and, if applicable, provide the name of the cohort or routinely collected database(s)*	54 (90%)	6 (10%)	0 (0%)	-
*Methods*						
Trial design	3^a^	CONSORT 2010: Description of trial design (such as parallel, factorial) including allocation ratio	17 (28%)	25 (42%)	18 (30%)	-
		Modified CONSORT-ROUTINE: Description of trial design (such as parallel, factorial) including allocation ratio, *that a cohort or routinely collected database(s) was used to conduct the trial (such as electronic health record, registry) and how the data were used within the trial (such as identification of eligible trial participants, trial outcomes)*	51 (85%)	4 (7%)	5 (8%)	-
Cohort or routinely collected database	ROUTINE-1	New CONSORT-ROUTINE: Name, if applicable, and description of the cohort or routinely collected database(s) used to conduct the trial, including information on the setting (such as primary care), locations, and dates, (such as periods of recruitment, follow-up, and data collection)	13 (22%)	40 (67%)	7 (12%)	-
	ROUTINE-2	New CONSORT-ROUTINE: Eligibility criteria for participants in the cohort or routinely collected database(s)	6 (10%)	8 (13%)	46 (77%)	-
	ROUTINE-3	New CONSORT-ROUTINE: State whether the study included person-level, institutional-level, or other data linkage across two or more databases and, if so, linkage techniques and methods used to evaluate completeness and accuracy of linkage	0 (0%)	5 (8%)	55 (92%)	-
Trial participants	4^a^	CONSORT 2010: Eligibility criteria for participants	50 (83%)	7 (12%)	3 (5%)	-
		Modified CONSORT-ROUTINE: Eligibility criteria for trial participants, *including information on how to access the list of codes and algorithms used to identify eligible participants, information on accuracy and completeness of data used to ascertain eligibility, and methods used to validate accuracy and completeness (e.g., monitoring, adjudication), if applicable*	1 (2%)	14 (23%)	27 (45%)	18 (30%)
	ROUTINE-4	New CONSORT-ROUTINE: Describe whether and how consent was obtained	35 (58%)	6 (10%)	19 (32%)	-
Outcomes	6^a^	CONSORT 2010: Completely defined pre-specified primary and secondary outcome measures, including how and when they were assessed	44 (73%)	8 (13%)	8 (13%)	-
		Modified CONSORT-ROUTINE: Completely defined pre-specified primary and secondary outcome measures, including how and when they were ascertained *and the cohort or routinely collected database(s) used to ascertain each outcome*	41 (68%)	8 (13%)	5 (8%)	6 (10%)
	ROUTINE-5	New CONSORT-ROUTINE: Information on how to access the list of codes and algorithms used to define or derive the outcomes from the cohort or routinely collected database(s) used to conduct the trial, information on accuracy and completeness of outcome variables, and methods used to validate accuracy and completeness (e.g., monitoring, adjudication), if applicable	3 (5%)	8 (13%)	42 (70%)	7 (12%)
Allocation concealment mechanism	9	CONSORT 2010: Mechanism used to implement the random allocation sequence (such as sequentially numbered containers), describing any steps taken to conceal the sequence until interventions were assigned^c^Modified CONSORT-ROUTINE: Mechanism used to implement the random allocation sequence *(such as embedding an automated randomiser within the cohort or routinely collected database(s)),* describing any steps taken to conceal the sequence until interventions were assigned^c^	16 (27%)	8 (13%)	36 (60%)	-
*Results*						
Participant flow (a diagram is strongly recommended)	13^a^	CONSORT 2010: For each group, the numbers of participants who were randomly assigned, received intended treatment, and were analysed for the primary outcome	45 (75%)	7 (12%)	8 (13%)	-
		Modified CONSORT-ROUTINE: For each group, the number of participants *in the cohort or routinely collected database(s) used to conduct the trial and the numbers screened for eligibility,* randomly assigned, *offered and accepted interventions (e.g., cohort multiple RCTs),* received intended treatment, and analysed for the primary outcome	6 (10%)	19 (32%)	15 (25%)	20 (33%)
*Discussion*						
Interpretation	22	CONSORT 2010: Interpretation consistent with results, balancing benefits and harms, and considering other relevant evidence	48 (80%)	11 (18%)	1 (2%)	-
		Modified CONSORT-ROUTINE: Interpretation consistent with results, balancing benefits and harms, and considering other relevant evidence, *including the implications of using data that were not collected to answer the trial research questions*	31 (52%)	18 (30%)	11 (18%)	-
*Other information*						
Funding	25	CONSORT 2010: Sources of funding and other support (such as supply of drugs), role of funders	25 (42%)	29 (48%)	6 (10%)	-
		Modified CONSORT-ROUTINE: Sources of funding and other support **for both the trial and the cohort or routinely collected database(s),** role of funders	3 (5%)	13 (22%)	44 (73%)	-

a For modified items, modifications are shown in italics. For those items, only portion modified was evaluated.

b Item numbers reflect numbers in original 2010 CONSORT checklist that were modified or new items. New items are designated by “ROUTINE”.

c 2010 and modified items not rated separately because modification was minor.

Of the eight items that were extension modifications, four were ‘adequately reported’ for most trials, including *EHR use in the abstract,* 90%, *Description of trial design,* 85%, *Outcomes,* 68% and *Interpretation,* 52%.

Funding was poorly reported (‘inadequately or not at all’) in the extension modification for *Funding (regarding cohort and routinely collected database),* 73%*.* Where applicable, the modified item *Eligibility criteria for trial participants (regarding information on codes/algorithms used to identify eligible participants, accuracy etc.)* was ‘inadequately or not at all’ reported in 64% (27 of 42) of cases. Likewise, where applicable, the modified item *Participant flow (enhanced for cohort and routinely collected data)* was ‘partially’ reported in 48% (19 of 40) of cases. The item *Allocation concealment mechanism* was not coded separately, as the modification was a clarification of the original item.

New items in CONSORT-ROUTINE: Of the five new items that were evaluated, only one was ‘adequately reported’ for most trials (>50%), *Informed consent* 58%. Three items were poorly (‘inadequately or not at all’) reported in most trials; these items included *Eligibility (for cohort or routinely collected database),* 92%, *Description of record linkage,* 77% and where applicable, *List of codes, monitoring and adjudication for outcomes*, 79% *(*42 of 53).

The new item *Description of the cohort or routinely collected database* was ‘partially reported’ in 67% of cases.

## 4 Discussion

Trials use EHRs in different ways, typically to deliver an intervention or to collect data, but also to identify participants; 44% of the trials in our review used the EHR to perform all three functions. In our sample, most RCTs using EHRs were conducted in the United States and in primary care, community or outpatient settings.

When a random sample of 60 publications (out of 183 identified) were compared to the CONSORT 2010 checklist items that had been modified in the 2021 CONSORT Extension for Trials Conducted Using Cohorts or Routinely Collected Data (CONSORT-ROUTINE) [14], only 4 (out of 8) were ‘adequately’ reported in most publications based on CONSORT 2010 content. This indicates that inadequate reporting precedes the development of the reporting extension. Perhaps most worrying, however, was the finding that the *Allocation concealment mechanism* (item 9) was reported ‘inadequately or not at all’ in 60% of cases, given the link between allocation concealment [23,24] with selection bias. Regarding the extension modifications of these 8 items, 4 were assessed as ‘adequately’ reported and 3 items were poorly reported in most trials. The remaining item was ‘partially’ reported in nearly half of trials.

For some modified items, the difference between the 2021 extension and 2010 items is marked. For items *Structured summary* and *Trial design*, which may overlap, the 2021 extension is reported much better, possibly due to mentioning the source of data. However, the opposite relationship is observed for *Participant flow, Interpretation* and *Funding*, which are reported much better for 2010 items, possibly due to a general lack of nuanced reporting of the genre.

When the sample of trials was assessed against new items in the 2021 CONSORT Extension for Trials Conducted Using Cohorts or Routinely Collected Data (CONSORT-ROUTINE), only 1 out of 5 items was ‘adequately’ reported in most trial publications. Three items were poorly reported, and the remaining item was ‘partially’ reported in most trials.

Similar reviews were conducted evaluating reporting in trials conducted using registries [25] and administrative datasets [26]. For most items, the reporting was much better for such trials than for trials conducted using EHRs. Nevertheless, there is considerable room for improvement in the reporting for these three types of trials conducted using routinely collected data, specifically regarding the broader issues of record linkage, eligibility for the routinely collected database(s), codes and algorithms related to outcome definitions, the allocation concealment mechanism, aspects of participant flow and funding source for the database(s).

The CONSORT checklist is a minimum standard for reporting. Clearly, there are issues specific to the use of routinely collected data that ideally would be reported in greater detail including: a general description of the EHR(s) used; throughput – eligibility and participant flow for the EHR(s) vs. the trial population; and for both data quality and completeness - the use of complex codes, algorithms and record linkage.

The poor reporting of these items matters because fundamentally it is difficult to assess the quality of trial conduct if reporting is substandard *per se,* and it is not surprising that new or modified items were not necessarily well reported. But there were also some encouraging signs with adequate and partial reporting of many important items, such as descriptions of trial design, the consent process, outcome ascertainment and interpretation.

The description of the use of the EHR within published RCTs was broadly consistent with a previous descriptive review of such trials [7] where most RCTs used the EHR as part of the intervention and to ascertain outcomes although a smaller proportion of RCTs used the EHR to identify eligible participants. The previous review was a descriptive study of RCTs that used an EHR, whilst our review used more comprehensive review methods, collected additional information on the characteristics of these RCTs and compared them by the level of randomization (cluster vs. individual), Although also evaluating the adequacy of reporting against existing and newly developed reporting standards.

We found that the transparency and completeness of reporting of RCTs conducted using EHRs to be sub-optimal, despite numerous reporting guidelines and publications. In particular, we found ongoing deficiencies in the adequacy of reporting key indicators of trial quality such as the mechanism for allocation concealment, clearly defined outcomes and the role of the funder [9,11,22,27,28]. The randomization process is fundamental to the integrity of the trial, and if poorly conducted, will increase the chances of a biased result. It is disappointing that such a fundamental component of any RCT is not well reported in these recent EHR based RCTs, despite the existence of evidence and recommendations [8,27,29,30].

This review provides a contemporary benchmark to assess the reporting of new or modified items included in the 2021 CONSORT Extension for Trials Conducted Using Routinely Collected Data (CONSORT-ROUTINE). As this reporting extension was not available at the time these trials were published, we did not expect these items to be particularly well reported. It is completely reasonable that the codes and algorithms used to identify participants or ascertain outcomes (and their completeness and accuracy) including details on record linkage were not well reported in the published EHR trials examined, because these items are not suggested in existing CONSORT reporting guidance or extensions, although they are in the RECORD reporting guidance for observational studies using routinely recorded data, published in 2015 [31]. These items are essential to understand the quality of the data [32], particularly the completeness and accuracy which are critical to internal validity and generalizability. Given that RCTs that use EHR data are increasingly common, this review highlights both the importance of transparency and completeness of reporting of specific aspects, and the deficiencies in reporting that exist.

Limitations include that the search may not have identified all published RCTs that used routinely collected data because the search strategy required the indication that electronic health records were involved either in the title, abstract or indexing. As a result, publications using a different term (e.g., RCHD) would not have been included. It is likely that this also resulted in several items being overestimated, because we may have selected examples of better reporting. Secondly, this review also only evaluated the reporting of new or modified items specific to trials conducted using EHRs and the corresponding CONSORT 2010 items. Therefore, it does not provide any information on more general aspects of trial reporting covered by other (non-modified) items from the CONSORT 2010. Thirdly, we performed an assessment of reporting quality, which is inherently subjective. To minimize this, we performed all extractions systematically, in duplicate, and disagreements were resolved by a third party; but we did not monitor inter-rater agreement.

## 5 Conclusion

EHRs are used in a variety of ways to help conduct RCTs. To improve the poor quality of reporting of these RCTs, the research community, journal editors, reviewers and funders should endorse and implement adherence to CONSORT 2010, and this 2021 extension for trials conducted using cohorts or routinely collected data. Improved awareness will improve reporting. The CONSORT explanation and elaboration document and checklist for RCTs using cohorts and routinely collected data will be made available on the EQUATOR website and published elsewhere [14].

## Availability of data and materials

Additional data beyond that reported in the main and supplementary materials can be requested from the corresponding author.

## Author contributions

Stephen J. McCall: Conceptualization, Protocol development & review, Project administration, Data curation, Formal analysis, Software, Investigation, Methodology, Validation, Writing – original draft. Mahrukh Imran: Conceptualization, Protocol review, Data curation, Investigation, Methodology, Project administration, Software, Validation, Writing – review & editing. Lars G. Hemkens: Conceptualization, Protocol review, Methodology, Supervision, Writing – review & editing. Kimberly McCord: Conceptualization, Data curation, Investigation, Methodology, Validation, Writing – review & editing. Linda Kwakkenbos: Conceptualization, Protocol review, Data curation, Funding acquisition, Investigation, Methodology, Supervision, Writing – review & editing. Margaret Sampson: Conceptualization, Methodology, Search, Writing – review & editing. Sena Jawad: Conceptualization, Data curation, Investigation, Methodology, Validation, Writing – review & editing. Merrick Zwarenstein: Conceptualization, Methodology, Writing – review & editing. Clare Relton: Conceptualization, Funding acquisition, Methodology, Writing – review & editing. Sinéad M. Langan: Conceptualization, Methodology, Writing – review & editing. David Moher: Conceptualization, Methodology, Writing – review & editing. Ole Fröbert: Conceptualization, Protocol review, Funding acquisition, Methodology, Writing – review & editing. Brett D. Thombs: Conceptualization, Protocol development & review, Data curation, Formal analysis, Funding acquisition, Methodology, Supervision, Writing – review & editing. Chris Gale: Conceptualization, Protocol development & review, Funding acquisition, Methodology, Supervision, Writing – review & editing.

Edmund Juszczak: Conceptualization, Protocol development & review, Funding acquisition, Methodology, Supervision, Writing – review & editing and approval of the manuscript as submitted.

## Acknowledgements

This part of the project was funded by the National Institute for Health Research (NIHR) CTU Support Funding scheme - Supporting efficient/innovative delivery of NIHR research (PI Juszczak, Co-PI Gale, supported salary of SM). The views expressed are those of the author(s) and not necessarily those of the NIHR or the Department of Health and Social Care. The development of CONSORT-ROUTINE and the present study were funded by grants from the Canadian Institutes of Health Research (PI Thombs, #PJT-156172; PIs Thombs and Kwakkenbos, #PCS-161863) and from the United Kingdom National Institute of Health Research (NIHR) Clinical Trials Unit Support Funding. Dr. Langan was supported by a Wellcome Senior Clinical Fellowship in Science (205039/Z/16/Z). Dr. Gale was supported by the United Kingdom Medical Research Council through a Clinician Scientist Fellowship. Dr. Thombs was supported by a Fonds de recherche du Québec - Santé researcher salary award and a Tier 1 Canada Research Chair. Dr. Moher was supported by a University Research Chair (uOttawa).

## Appendix Supplementary materials

Image, application 1
