# Supplementary material for: Reporting transparency and completeness in trials: Paper 4 - reporting of randomised controlled trials conducted using routinely collected electronic records – room for improvement
Source: J Clin Epidemiol. 2022 Jan;141:198–209. doi: 10.1016/j.jclinepi.2021.09.011 (PMC8982641; doi:10.1016/j.jclinepi.2021.09.011)
Supplement: Supplementary file 1 [file mmc1.pdf]

Appendices

Appendix 1. Electronic search strategy

Adapted from published protocol (15). Searches were run in both MEDLINE and Cochrane Methodology Register simultaneously. As an example, in the registries search, lines 1-11 are the MEDLINE search and lines 12-15 are tailored for the Cochrane Methodology Register. The final lines of each search isolate the records from each database, combine them so duplicate records can be removed, then isolate the remaining records so they can be downloaded and imported into Reference Manager using customized import filters.

Searching for RCTs conducted using Electronic Health Records

1. randomized controlled trial.pt.
2. controlled clinical trial.pt.
3. randomi?ed.ab.
4. placebo.ab.
5. randomly.ab.
6. clinical trials as topic.sh.
7. trial.ti.
8. or/1-7
9. exp animals/ not humans.sh.
10. 8 not 9
11. exp Electronic Health Records/
12. (EHR or electronic health record*).ab,kf,ti.
13. (EMR or electronic medical record*).ab,kf,ti.
14. (PHR or personal health record*).ab,kf,ti.
15. (EPR or electronic patient record*).ab,kf,ti.
16. exp Health Records, Personal/
17. or/11-16
18. 10 and 17
19. limit 18 to yr="2007 - 2018"
20. (Electronic health record or electronic health records or EHR).ti,ab,kw.
21. (Electronic medical record or electronic medical records or EMR).ti,ab,kw.
22. (Electronic patient record or electronic patient records or EPR).ti,ab,kw.
23. or/20-22
24. limit 23 to yr="2007 - 2018"
25. 19 use medall
26. 24 use clcmr
27. 25 or 26
28. remove duplicates from 27
29. 28 use medall
30. 28 use clcmr

Appendix 2. Inclusion criteria algorithm

Eligible RCTs have to use EHRs. Publications that report (1) issues related to methods or reporting of EHR-based RCTs, or (2) a protocol from an RCT conducted using EHRs are excluded. If the RCT involved non-human subjects, it is excluded. Only RCTs that use EHRs as a source of data for conducting the trial, including activities such as identifying eligible participants for the trial or as an intervention or collecting trial outcomes, are eligible. To be included in the review, a publication had to meet one of the following criteria:

1. The EHR is used for identifying eligible participants. If the publication describes a trial in which the EHR was used to identify eligible trial participants, it will be included.

2. The EHR can be used as an intervention or component of an intervention, such as alerting physicians to deliver a screening procedure for some patients, and different trial arms may involve different access to EHR functions. If the publication describes an RCT that used the EHR as part of the intervention, it will be included.

3. The EHR is used to ascertain health outcomes. If the publication describes a trial that links to an EHR to ascertain health outcomes, as trial endpoints, it will be included.

Appendix 3. Coding manual for the completeness and transparency of reporting

Section/Topic	Item No.	CONSORT 2010 Item	CONSORT-ROUTINE	Adequately reported	Partially reported	Inadequately or not reported	Not applicable
Title and abstract							
		Structured summary of trial design, methods, results, and conclusions (for specific guidance see CONSORT for abstracts)		Did the authors clearly describe a (1) structured summary of (2) trial design, (3) methods, (4) results, and (5) conclusions	Did the authors only report one, two, three or four element(s) of this item and not all five elements of the item?	Did the authors not describe a structured summary of trial design, methods, results and conclusions?	
	1b		Structured summary of trial design, methods, results, and conclusions (for specific guidance see CONSORT for abstracts). Specify that a cohort or routinely collected data were used to conduct the trial and, if applicable, provide the name of the cohort or routinely collected database(s) (Modified)	Did the authors specify that a cohort or routinely collected data were used to conduct the trial?	Did the authors describe methods that would typically require routinely collected data for components of the trials but not specify they used routinely collected data?	Did the authors not specify that routinely collected data were used to conduct the trial?	
Introduction							
Trial design	3a	Description of trial design (such as parallel, factorial) including allocation ratio		Did the authors clearly describe the trial design including allocation ratio?	All other cases, where applicable.	Did the authors not describe the trial design including allocation ratio?	
			Description of trial design (such as parallel, factorial) including allocation ratio, that a cohort or routinely collected database(s) was used to conduct the trial (such as electronic health record, registry) and how the data were used within the trial (such as identification of eligible trial participants, trial outcomes) (Modified)	Did the authors clearly mention the (1) routinely collected database(s) that were used within the trial and (2) how the data were used within the trial (i.e. identification of participants, outcome measurement, other)?	Did the authors only report one element of this item and not both elements of the item?	Did the authors not describe the routinely collected database(s) that were used within the trial and not describe how the data were used within the trial (i.e. identification of participants, outcome measurement, other)?	
Cohort or routinely collected data							
	ROUTINE-		Name, if applicable, and description of	Did the authors clearly (1)	Did the authors only	Did the authors not name	

	1		the cohort or routinely collected database(s) used to conduct the trial, including information on the setting (such as primary care), locations, and dates, (such as periods of recruitment, follow-up, and data collection) (New)	name and (2) describe the routinely collected database(s) and (3) provide information on the setting, locations, and relevant dates (e.g. periods of recruitment, follow-up, and data collection)?	report one or two element(s) of this item and not all three elements of the item?	and describe the routinely collected database(s) and not provide information on the setting, locations, and relevant dates (e.g. periods of recruitment, follow-up, and data collection)?	
	ROUTINE-2		Eligibility criteria for participants in the cohort or routinely collected database(s) (New)	Did the authors clearly describe the eligibility criteria for the routinely collected database(s)?	All other cases, where applicable.	Did the authors not describe all eligibility criteria for the routinely collected database(s)?	
	ROUTINE-3		State whether the study included person-level, institutional-level, or other data linkage across two or more databases and, if so, linkage techniques and methods used to evaluate completeness and accuracy of linkage (New)	Did the authors clearly state whether the study included (1) person-level, institutional-level, or other data linkage across two or more databases and (2) the methods of linkage and (3) methods used to evaluate completeness and accuracy of linkage?	Did the authors only report one element of this item and not all three elements of the item?	Did the authors not state whether the study included person-level, institutional-level, or other data linkage across two or more databases and not state the methods of linkage and methods used to evaluate completeness and accuracy of linkage?	
Trial participants	4a	Eligibility criteria for participants		Did the authors clearly describe the eligibility for the trial participants?	All other cases, where applicable.	Did the authors not describe all eligibility criteria for the trial participants?	
			Eligibility criteria for trial participants, including information on how to access the list of codes and algorithms used to identify eligible participants, information on accuracy and completeness of data used to ascertain eligibility, and methods used to validate accuracy and completeness (e.g., monitoring, adjudication), if applicable (Modified)	Did the authors provide information on (1) how to access the lists of codes and algorithms used to identify participants, including (2) methods used to assess accuracy and completeness, if applicable?	Did the authors only report one element of this item and not both elements of the item?	Did the authors not provide information on how to access the lists of codes and algorithms used to identify participants, and not provide the methods used to assess accuracy and completeness?	The trial did not use routinely collected data to identify participants
	ROUTINE-4		Describe whether and how consent was obtained (New)	Did the authors describe clearly whether and how consent was obtained?	All other cases, where applicable.	Did the authors not describe whether and how consent was obtained?	
Outcomes	6a	Completely defined pre-specified primary and secondary outcome		Did the authors clearly define the pre-specified primary and secondary	Did the authors only define the pre-specified primary and	Did the authors not define the pre-specified primary and secondary outcome measures	

		measures, including how and when they were assessed		outcome measures, including how and when they were assessed?	secondary outcome measures but not how and when they were assessed, or did they describe how and when outcomes were assessed but not the measures?	and not define how and when they were assessed?	
			Completely defined pre-specified primary and secondary outcome measures, including how and when they were ascertained and the cohort or routinely collected database(s) used to ascertain each outcome (Modified)	Did the authors clearly describe the routinely collected database(s) used to ascertain each outcome?	All other cases, where applicable.	Did the authors not describe the routinely collected database(s) used to ascertain each outcome?	The trial did not use routinely collected data to ascertain the outcome
	ROUTINE-5		Information on how to access the list of codes and algorithms used to define or derive the outcomes from the cohort or routinely collected database(s) used to conduct the trial, information on accuracy and completeness of outcome variables, and methods used to validate accuracy and completeness (e.g., monitoring, adjudication), if applicable (New)	Did the authors clearly (1) describe information on how to access the list of codes and algorithms used to define or derive the outcomes from the routinely collected database(s), (2) including methods used to assess accuracy and completeness?	Did the authors only report one element of this item and not both elements of the item?	Did the authors not describe information on how to access the list of codes and algorithms used to define or derive the outcomes from the routinely collected database(s), and not describe the methods used to assess accuracy and completeness?	The trial did not use routinely collected data to ascertain the outcome
Allocation concealment mechanism	9	Mechanism used to implement the random allocation sequence (such as sequentially numbered containers), describing any steps taken to conceal the sequence until interventions were assigned	Mechanism used to implement the random allocation sequence (such as embedding an automated randomiser within the cohort or routinely collected database(s)), describing any steps taken to conceal the sequence until interventions were assigned (Modified)	Did the authors clearly describe the mechanism used to implement the random allocation sequence (such as embedding an automated randomiser within the cohort or routinely collected database(s)), describing any steps taken to conceal the sequence until interventions were assigned?	All other cases, where applicable	Did the authors not describe the mechanism used to implement the random allocation sequence (such as embedding an automated randomiser within the cohort or routinely collected database(s)), describing any steps taken to conceal the sequence until interventions were assigned?	

Participant flow (a diagram is strongly recommended)	13a	For each group, the numbers of participants who were randomly assigned, received intended treatment, and were analysed for the primary outcome		Did the authors define clearly for each group, (1) the number of participants who were randomly assigned, (2) received intended treatment and (3) were analysed for the primary outcome?	Did the authors only report one or two elements of this item and not all three elements of the item or only presented this information for one group?	Did the authors not describe clearly for each group, the number of participants who were randomly assigned, and not received intended treatment and not were analysed for the primary outcome?	
			For each group, the number of participants in the cohort or routinely collected database(s) used to conduct the trial and the numbers screened for eligibility, randomly assigned, offered and accepted interventions (e.g., cohort multiple RCTs), received intended treatment, and analysed for the primary outcome (Modified)	Did the authors clearly define, for each group, the number of participants in the routinely collected database(s) used to conduct the trial and the numbers screened for eligibility, randomly assigned, received intended treatment, and analysed for the primary outcome?	Did the authors only report some, but not all, elements of this item?	Did the authors not define, for each group, the number of participants in the routinely collected database(s) used to conduct the trial and not define the numbers screened for eligibility, randomly assigned, received intended treatment, and analysed for the primary outcome	
Discussion							
Interpretation	22	Interpretation consistent with results, balancing benefits and harms, and considering other relevant evidence		Did the authors clearly provide an interpretation consistent with results, balancing benefits and harms, and considering other relevant evidence?	All other cases, where applicable	Did the authors not provide an interpretation consistent with results, balancing benefits and harms, and considering other relevant evidence?	
			Interpretation consistent with results, balancing benefits and harms, and considering other relevant evidence, including the implications of using data that were not collected to answer the trial research questions (Modified)	Did the authors describe the implications of using data that were not collected to answer the trial research questions?<br><br>Note: the authors have to report information about the issues of using routine data.	All other cases, where applicable	Did the authors not describe the implications of using data that were not collected to answer the trial research questions?	
Other information							
Funding	25	Sources of funding and other support (such as supply of drugs), role of funders		Did the authors clearly describe the sources of funding and the role of funders?	All other cases, where applicable	Did the authors not describe the sources of funding and other support for the trial and the role of the funders?	

			Sources of funding and other support for both the trial and the cohort or routinely collected database(s), role of funders (Modified)	Did the authors clearly describe the sources of funding for the database(s) and trial and the role of the funder of the trial?	Did the authors only report some, but not all, elements of this item?	Did the authors not describe the sources of funding for routinely collected database(s) and trial and not describe the role of the funder of the trial?	

Appendix 4. References of the 60 RCT publications assessed for completeness and transparency of reporting

1. Abdel-Kader K, Fischer GS, Li J, Moore CG, Hess R, Unruh ML. Automated clinical reminders for primary care providers in the care of CKD: a small cluster-randomized controlled trial. American Journal of Kidney Diseases. 2011;58:894
2. Adams WG, Phillips BD, Bacic JD, Walsh KE, Shanahan CW, Paasche-Orlow MK. Automated conversation system before pediatric primary care visits: a randomized trial. Pediatrics. 2014;134:e691
3. Amemori M, Virtanen J, Korhonen T, Kinnunen TH, Murtomaa H. Impact of educational intervention on implementation of tobacco counselling among oral health professionals: a cluster-randomized community trial. Community Dentistry & Oral Epidemiology. 2013;41:120
4. Arts DL, bu-Hanna A, Medlock SK, van Weert HC. Effectiveness and usage of a decision support system to improve stroke prevention in general practice: A cluster randomized controlled trial. PLoS ONE. 2017;12:e0170974
5. Awdishu L, Coates CR, Lyddane A, Tran K, Daniels CE, Lee J, et al. The impact of real-time alerting on appropriate prescribing in kidney disease: a cluster randomized controlled trial. Journal of the American Medical Informatics Association. 2016;23:609
6. Beurden van, Anderson P, Akkermans RP, Grol RP, Wensing M, Laurant MG. Involvement of general practitioners in managing alcohol problems: a randomized controlled trial of a tailored improvement programme. Addiction. 2012;107:1601
7. Bleijenberg N, Drubbel I, Schuurmans MJ, Dam HT, Zuithoff NP, Numans ME, et al. Effectiveness of a Proactive Primary Care Program on Preserving Daily Functioning of Older People: A Cluster Randomized Controlled Trial. Journal of the American Geriatrics Society. 2016;64:1779
8. Carroll AE, Biondich PG, Anand V, Dugan TM, Sheley ME, Xu SZ, et al. Targeted screening for pediatric conditions with the CHICA system. Journal of the American Medical Informatics Association. 2011;18:485
9. Chiche L, Brescianini A, Mancini J, Servy H, Durand JM. Evaluation of a prototype electronic personal health record for patients with idiopathic thrombocytopenic purpura. Patient preference & adherence. 2012;6:725
10. Cho SW, Wee JH, Yoo S, Heo E, Ryu B, Kim Y, et al. Effect of Lifestyle Modification Using a Smartphone Application on Obesity With Obstructive Sleep Apnea: A Short-term, Randomized Controlled Study. Clinical & Experimental Otorhinolaryngology. 2018;11:192
11. Conroy MB, Bryce CL, McTigue KM, Tudorascu D, Gibbs BB, Comer D, et al. Promoting weight maintenance with electronic health record tools in a primary care setting: Baseline results from the MAINTAIN-pc trial. Contemporary Clinical Trials. 2017;54:60
12. Denig P, Schuling J, Haaijer-Ruskamp F, Voorham J. Effects of a patient oriented decision aid for prioritising treatment goals in diabetes: pragmatic randomised controlled trial. BMJ. 2014;349:g5651
13. Dregan A, van Staa TP, McDermott L, McCann G, Ashworth M, Charlton J, et al. Point-of-care cluster randomized trial in stroke secondary prevention using electronic health records. Stroke. 2014;45:2066
14. Enns E, Rhemtulla R, Ewa V, Fruetel K, Holroyd-Leduc JM. A controlled quality improvement trial to reduce the use of physical restraints in older hospitalized adults. Journal of the American Geriatrics Society. 2014;62:541

15. Feder G, Davies RA, Baird K, Dunne D, Eldridge S, Griffiths C, et al. Identification and Referral to Improve Safety (IRIS) of women experiencing domestic violence with a primary care training and support programme: a cluster randomised controlled trial. Lancet. 2011;378:1788
16. Ford I, Murray H, McCowan C, Packard CJ. Long-Term Safety and Efficacy of Lowering Low-Density Lipoprotein Cholesterol With Statin Therapy: 20-Year Follow-Up of West of Scotland Coronary Prevention Study. Circulation. 2016;133:1073
17. Fu SS, van RynM, Sherman SE, Burgess DJ, Noorbaloochi S, Clothier B, et al. Proactive tobacco treatment and population-level cessation: a pragmatic randomized clinical trial. JAMA Internal Medicine. 2014;174:671
18. Gallagher LM, Gardner V, Bates D, Mason S, Nemecek J, DiFiore JB, et al. Impact of Music Therapy on Hospitalized Patients Post-Elective Orthopaedic Surgery: A Randomized Controlled Trial. Orthopaedic Nursing. 2018;37:124
19. Gerber JS, Prasad PA, Fiks AG, Localio AR, Grundmeier RW, Bell LM, et al. Effect of an outpatient antimicrobial stewardship intervention on broad-spectrum antibiotic prescribing by primary care pediatricians: a randomized trial. JAMA. 2013;309:2345
20. Ghadieh AS, Hamadeh GN, Mahmassani DM, Lakkis NA. The effect of various types of patients' reminders on the uptake of pneumococcal vaccine in adults: A randomized controlled trial. Vaccine. 2015;33:5868
21. Gidding LG, Spigt M, Winkens B, Herijgers O, Dinant GJ. PsyScan e-tool to support diagnosis and management of psychological problems in general practice: a randomised controlled trial. British Journal of General Practice. 2018;68:e18
22. Graumlich JF, Wang H, Madison A, Wolf MS, Kaiser D, Dahal K, et al. Effects of a Patient-Provider, Collaborative, Medication-Planning Tool: A Randomized, Controlled Trial. Journal of Diabetes Research. 2016;2016:2129838
23. Green BB, Anderson ML, Wang CY, Vernon SW, Chubak J, Meenan RT, et al. Results of nurse navigator follow-up after positive colorectal cancer screening test: a randomized trial. Journal of the American Board of Family Medicine: JABFM. 2014;27:789
24. Greenwood DA, Blozis SA, Young HM, Nesbitt TS, Quinn CC. Overcoming Clinical Inertia: A Randomized Clinical Trial of a Telehealth Remote Monitoring Intervention Using Paired Glucose Testing in Adults With Type 2 Diabetes. Journal of Medical Internet Research. 2015;17:e178
25. Gupta A, Gholami P, Turakhia MP, Friday K, Heidenreich PA. Clinical reminders to providers of patients with reduced left ventricular ejection fraction increase defibrillator referral: a randomized trial. Circulation: Heart Failure. 2014;7:140
26. Haas JS, Linder JA, Park ER, Gonzalez I, Rigotti NA, Klinger EV, et al. Proactive tobacco cessation outreach to smokers of low socioeconomic status: a randomized clinical trial. JAMA Internal Medicine. 2015;175:218
27. Hanson AL, Schunk JE, Corneli HM, Soprano JV. A Randomized Controlled Trial of Positioning for Lumbar Puncture in Young Infants. Pediatric emergency care. 2016;32:504
28. Harle CA, Marlow NM, Schmidt SO, Shuster JJ, Listhaus A, Fillingim RB, et al. The effect of EHR-integrated patient-reported outcomes on satisfaction with chronic pain care. American Journal of Managed Care. 2016;22:e403
29. Holmes JF, Freilich J, Taylor SL, Buettner D. Electronic alerts for triage protocol compliance among emergency department triage nurses: a randomized controlled trial. Nursing research. 2015;64:226
30. Holt TA, Dalton A, Marshall T, Fay M, Qureshi N, Kirkpatrick S, et al. Automated Software System to Promote Anticoagulation and Reduce Stroke Risk: Cluster-Randomized Controlled Trial. Stroke. 2017;48:787

31. Jouni H, Haddad RA, Marroush TS, Brown SA, Kruisselbrink TM, Austin EE, et al. Shared decision-making following disclosure of coronary heart disease genetic risk: results from a randomized clinical trial. Journal of Investigative Medicine. 2017;65:681
32. Kharbanda EO, Asche SE, Sinaiko AR, Ekstrom HL, Nordin JD, Sherwood NE, et al. Clinical Decision Support for Recognition and Management of Hypertension: A Randomized Trial. Pediatrics. 2018;141: e20172954
33. Kortteisto T, Raitanen J, Komulainen J, Kunnamo I, Makela M, Rissanen P, et al. Patient-specific computer-based decision support in primary healthcare--a randomized trial. Implementation Science. 2014;9:15
34. Lewis CL, Adams J, Tai-Seale M, Huang Q, Knowles SB, Nielsen ME, et al. A Randomized Controlled Effectiveness Trial for PSA Screening Decision Support Interventions in Two Primary Care Settings. Journal of General Internal Medicine. 2015;30:810
35. Lewis CL, Brenner AT, Griffith JM, Moore CG, Pignone MP. Two controlled trials to determine the effectiveness of a mailed intervention to increase colon cancer screening. North Carolina medical journal. 2012;73:93
36. Liebschutz JM, Xuan Z, Shanahan CW, LaRochelle M, Keosaian J, Beers D, et al. Improving Adherence to Long-term Opioid Therapy Guidelines to Reduce Opioid Misuse in Primary Care: A Cluster-Randomized Clinical Trial. JAMA Internal Medicine. 2017;177:1265
37. Ma J, Yank V, Xiao L, Lavori PW, Wilson SR, Rosas LG, et al. Translating the Diabetes Prevention Program lifestyle intervention for weight loss into primary care: a randomized trial. JAMA Internal Medicine. 2013;173:113
38. Margolis KL, Asche SE, Bergdall AR, Dehmer SP, Groen SE, Kadrmas HM, et al. Effect of home blood pressure telemonitoring and pharmacist management on blood pressure control: a cluster randomized clinical trial. JAMA. 2013;310:46
39. Marino M, Bailey SR, Gold R, Hoopes MJ, O'Malley JP, Huguet N, et al. Receipt of Preventive Services After Oregon's Randomized Medicaid Experiment. American Journal of Preventive Medicine. 2016;50:161
40. McCarren M, Furmaga E, Jackevicius CA, Sahay A, Coppler TL, Katzianer J, et al. Improvement of guideline beta-blocker prescribing in heart failure: a cluster-randomized pragmatic trial of a pharmacy intervention. Journal of cardiac failure. 2013;19:525
41. McGinn TG, McCullagh L, Kannry J, Knaus M, Sofianou A, Wisnivesky JP, et al. Efficacy of an evidence-based clinical decision support in primary care practices: a randomized clinical trial. JAMA Internal Medicine. 2013;173:1584
42. Otsuka SH, Tayal NH, Porter K, Embi PJ, Beatty SJ. Improving herpes zoster vaccination rates through use of a clinical pharmacist and a personal health record. American Journal of Medicine. 2013;126:832
43. Patja K, Absetz P, Auvinen A, Tokola K, Kyto J, Oksman E, et al. Health coaching by telephony to support self-care in chronic diseases: clinical outcomes from The TERVA randomized controlled trial. BMC Health Services Research. 2012;12:147
44. Rathlev N, Almomen R, Deutsch A, Smithline H, Li H, Visintainer P. Randomized Controlled Trial of Electronic Care Plan Alerts and Resource Utilization by High Frequency Emergency Department Users with Opioid Use Disorder. The Western Journal of Emergency Medicine. 2016;17:28
45. Ryan AM, McCullough CM, Shih SC, Wang JJ, Ryan MS, Casalino LP. The intended and unintended consequences of quality improvement interventions for small practices in a community-based electronic health record implementation project. Medical care. 2014;52:826

46. Semler MW, Wanderer JP, Ehrenfeld JM, Stollings JL, Self WH, Siew ED, et al. Balanced Crystalloids versus Saline in the Intensive Care Unit. The SALT Randomized Trial. American Journal of Respiratory & Critical Care Medicine. 2017;195:1362
47. Sequist TD, Morong SM, Marston A, Keohane CA, Cook EF, Orav EJ, et al. Electronic risk alerts to improve primary care management of chest pain: a randomized, controlled trial. Journal of General Internal Medicine. 2012;27:438
48. Sherman SE, Krebs P, York LS, Cummins SE, Kuschner W, Guvenc-Tuncturk S, et al. Telephone care co-ordination for tobacco cessation: randomised trials testing proactive versus reactive models. Tobacco control. 2018;27:78
49. Skinner CS, Halm EA, Bishop WP, Ahn C, Gupta S, Farrell D, et al. Impact of Risk Assessment and Tailored versus Nontailored Risk Information on Colorectal Cancer Testing in Primary Care: A Randomized Controlled Trial. Cancer Epidemiology, Biomarkers & Prevention. 2015;24:1523
50. Sterling S, Kline-Simon AH, Jones A, Satre DD, Parthasarathy S, Weisner C. Specialty addiction and psychiatry treatment initiation and engagement: Results from an SBIRT randomized trial in pediatrics. Journal of substance abuse treatment. 2017;82:48
51. Sterling S, Kline-Simon AH, Weisner C, Jones A, Satre DD. Pediatrician and Behavioral Clinician-Delivered Screening, Brief Intervention and Referral to Treatment: Substance Use and Depression Outcomes. Journal of Adolescent Health. 2018;62:390
52. Tajmir S, Raja AS, Ip IK, Andruchow J, Silveira P, Smith S, et al. Impact of Clinical Decision Support on Radiography for Acute Ankle Injuries: A Randomized Trial. The Western Journal of Emergency Medicine. 2017;18:487
53. Tang JW, Kushner RF, Cameron KA, Hicks B, Cooper AJ, Baker DW. Electronic tools to assist with identification and counseling for overweight patients: a randomized controlled trial. Journal of General Internal Medicine. 2012;27:933
54. Vollmer WM, Feldstein A, Smith DH, Dubanoski JP, Waterbury A, Schneider JL, et al. Use of health information technology to improve medication adherence. American Journal of Managed Care. 2011;17:SP79
55. Vollmer WM, Owen-Smith AA, Tom JO, Laws R, Ditmer DG, Smith DH, et al. Improving adherence to cardiovascular disease medications with information technology. American Journal of Managed Care. 2014;20:SP502
56. Yi SS, Tabaei BP, Angell SY, Rapin A, Buck MD, Pagano WG, et al. Self-blood pressure monitoring in an urban, ethnically diverse population: a randomized clinical trial utilizing the electronic health record. Circulation: Cardiovascular Quality & Outcomes. 2015;8:138
57. Young LA, Buse JB, Weaver MA, Vu MB, Mitchell CM, Blakeney T, et al. Glucose Self-monitoring in Non-Insulin-Treated Patients With Type 2 Diabetes in Primary Care Settings: A Randomized Trial. JAMA Internal Medicine. 2017;177:920
58. Zeiger RS, Schatz M, Li Q, Solari PG, Zazzali JL, Chen W. Real-time asthma outreach reduces excessive short-acting beta2-agonist use: a randomized study. The Journal of Allergy & Clinical Immunology in Practice. 2014;2:445
59. Zera CA, Bates DW, Stuebe AM, Ecker JL, Seely EW. Diabetes Screening Reminder for Women With Prior Gestational Diabetes: A Randomized Controlled Trial. Obstetrics & Gynecology. 2015;126:109
60. Zimmerman RK, Raviotta JM, Nowalk MP, Moehling KK, Reis EC, Humiston SG, et al. Using the 4 PillarsTM Practice Transformation Program to increase adolescent human papillomavirus, meningococcal, tetanus-diphtheria-pertussis and influenza vaccination. Vaccine. 2017;35:6180
